# Characterization of 7A7, an anti-mouse EGFR monoclonal antibody proposed to be the mouse equivalent of cetuximab

**DOI:** 10.18632/oncotarget.24242

**Published:** 2018-01-13

**Authors:** Xuzhi He, Jazmina L. Cruz, Shannon Joseph, Nicola Pett, Hui Yi Chew, Zewen K. Tuong, Satomi Okano, Gabrielle Kelly, Margaret Veitch, Fiona Simpson, James W. Wells

**Affiliations:** ^1^ The University of Queensland Diamantina Institute, Faculty of Medicine, University of Queensland, Translational Research Institute, Brisbane, QLD, Australia; ^2^ Queensland Head and Neck Cancer Centre, Princess Alexandra Hospital, Brisbane, QLD, Australia

**Keywords:** 7A7, cetuximab, mouse epidermal growth factor receptor, IgG1

## Abstract

The Epidermal Growth Factor Receptor (EGFR) is selectively expressed on the surface of numerous tumours, such as non-small cell lung, ovarian, colorectal and head and neck carcinomas. EGFR has therefore become a target for cancer therapy. Cetuximab is a chimeric human/mouse monoclonal antibody (mAb) that binds to EGFR, where it both inhibits signaling and induces cell death by antibody-dependent cell mediated cytotoxicity (ADCC). Cetuximab has been approved for clinical use in patients with head and neck squamous cell carcinoma (HNSCC) and colorectal cancer. However, only 15-20% patients benefit from this drug, thus new strategies to improve cetuximab efficiency are required. We aimed to develop a reliable and easy preclinical mouse model to evaluate the efficacy of EGFR-targeted antibodies and examine the immune mechanisms involved in tumour regression. We selected an anti-mouse EGFR mAb, 7A7, which has been reported to be “mouse cetuximab” and to exhibit similar properties to its human counterpart. Unfortunately, we were unable to reproduce previous results obtained with the 7A7 mAb. In our hands, 7A7 failed to recognize mouse EGFR, both in native and reducing conditions. Moreover, *in vivo* administration of 7A7 in an EGFR-expressing HPV38 tumour model did not have any impact on tumour regression or animal survival. We conclude that 7A7 does not recognize mouse EGFR and therefore cannot be used as the mouse equivalent of cetuximab use in humans. As a number of groups have spent effort and resources with similar issues we feel that publication is a responsible approach.

## INTRODUCTION

The Epidermal Growth Factor Receptor (EGFR) is a 170-kDa protein that belongs to the ErbB family of receptor tyrosine kinases. It is composed of an extracellular ligand-binding domain, a single hydrophobic transmembrane region and an intracellular domain with intrinsic kinase activity that regulates many developmental, metabolic, and physiological processes [1, 2]. EGFR overexpression is associated with the development of several tumour malignancies, such as non-small cell lung cancer, ovarian cancer, colorectal cancer and head and neck cancer [3]. Different EGFR signaling output pathways, such as the mitogen-activated protein kinases (MAPK) and the phosphoinositide 3-kinase/Akt pathway, result in cell proliferation, migration and modulation of ion channels which may contribute to tumour invasion, metastasis and progression [4]. Therefore, EGFR has been regarded as a central target for cancer therapy.

There are several EGFR-targeted drugs currently used in the clinic including therapeutic monoclonal antibodies (mAbs), which bind to the extracellular domain of EGFR, and small molecule inhibitors that target the EGFR signaling cascade, such as tyrosine kinase inhibitors (TKIs) [5]. Cetuximab is a chimeric human/mouse mAb that binds to the extracellular domain of EGFR and is approved by the FDA for the treatment of colorectal cancer with KRAS WT status and HNSCC [6]. Even though the majority of HNSCC patient tumours express EGFR (∼98%), only approximately 15-20% of patients respond positively and benefit from this treatment [7, 8]. Previous studies have shown that cetuximab can trigger the innate or adaptive immune system, but the mechanisms are still unclear and need to be further studied [9–12].

In order to develop a reliable and easy preclinical mouse model to allow further analysis of immune system involvement in anti-EGFR mAb treatment, we selected an anti-mouse EGFR monoclonal antibody (7A7) to model cetuximab treatment. 7A7 was first produced and published in 2004 [13] where it was suggested to be a valuable antibody for EGFR-based therapeutic preclinical studies in mice. The original study showed 7A7 could successfully recognize mouse EGFR expression both in cells and tissue samples by fluorescence-activated cell sorting (FACS), Western blot (WB) and immunohistochemistry [13]. 7A7 was also described to prolong survival and show anti-metastatic effects in a D122 mouse tumour model [14]. These results suggested that 7A7 was a good candidate for EGFR targeted preclinical studies in mice, which led to its use in our study.

The objective of this study was to create an *in vitro* and *in vivo* pre-clinical platform with which to model cetuximab, using the monoclonal antibody 7A7. This would enable investigation of the impact that these therapies have on immune system activation and allow the assessment of underlying immune mechanisms of tumour rejection. Thus, in order to develop our mouse experiments, we tested the capacity of 7A7 to bind murine EGFR and to induce tumour regression. As a control, we parallel tested 7A7 Fc Silent, which contains key mutations that abrogate binding of Fc receptors, abolishing the antibody mediated cytotoxicity (ADCC) effector function of 7A7. Theoretically, both 7A7 and 7A7 Fc Silent should recognize mouse EGFR and cross-react with human EGFR.

We demonstrate that neither 7A7 nor 7A7 Fc Silent specifically recognize either mouse or human EGFR. 7A7 was unable to impact tumour growth in an EGFR-expressing HPV38 transplantable SCC tumour model. Our study is an exhaustive *in vitro* and *in vivo* characterization of the 7A7 monoclonal antibody. We trust our results can allow researchers to make an informed decision when considering 7A7 for EGFR-targeted preclinical studies in mice.

## RESULTS

### EGFR mRNA expression in human and murine cell lines

**Figure 1:**
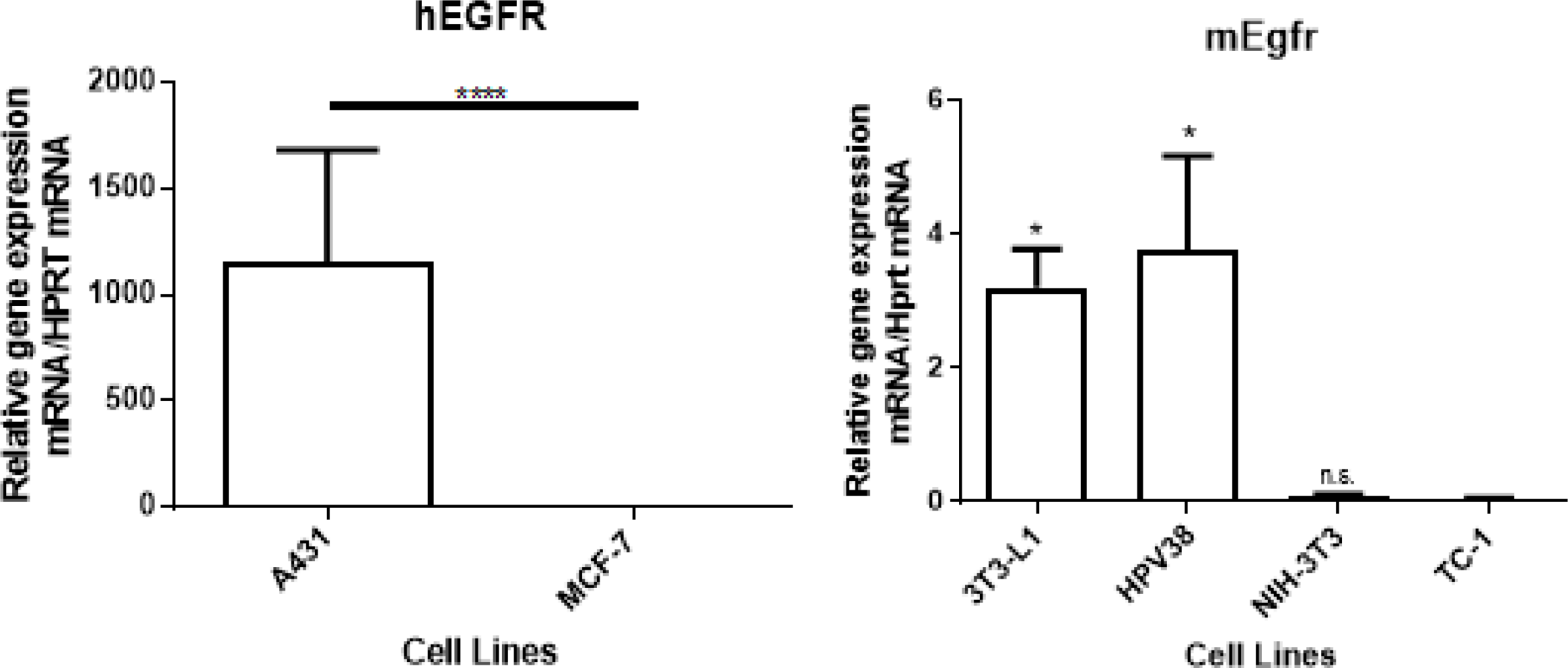
EGFR mRNA expression *in vitro* in 2 human cell lines (A431 and MCF-7) and 4 murine cell lines (3T3-L1, HPV38, NIH-3T3, and TC-1) (**A**) A431 expressed more than 1 thousand-fold EGFR compared to MCF-7 cells. (**B**) 3T3-L1 and HPV38 expressed detectable levels of Egfr. NIH-3T3 and TC-1 did not express detectable levels of Egfr. The *p*-value was determined using (A) unpaired student’s *t*-test or (B) one-way ANOVA analysis, followed by a post-hoc Tukey’s multiple comparisons test (data compared with TC-1), *n* = 3 (^*^p < 0.05, ^****^p < 0.0001, n.s: not significant). Data shown is representative of one experiment of three with similar results.

We selected 6 cell lines to analyze the specificity of 7A7 to detect graded levels of mouse and human EGFR. We quantified EGFR mRNA by quantitative RT-PCR in 2 human cell lines (A431 and MCF-7) and 4 murine cell lines (3T3-L1, NIH-3T3, HPV38, and TC-1). The human cell line A431 expressed more than one thousand-fold EGFR at the mRNA level when compared to MCF-7 cells (Figure 1A), in line with previously published data [15].

Based on a previous report describing the presence of Egfr expression in 3T3-L1 cells [16], we selected 3T3-L1 cells as a comparator for Egfr expression in our cohort of murine cell lines. As shown in Figure 1B, Egfr mRNA levels varied widely among the 4 cell types tested. A SCC cell line that we developed in-house, HPV38, showed high Egfr mRNA expression similar to that of 3T3-L1 cells. In contrast, NIH-3T3 and TC-1 cells showed no significant evidence of Egfr mRNA expression, however, TC-1 cells are known to be tumourigenic when injected into mice (of relevance later) and hence TC-1 cells were selected for further study in subsequent experiments.

### Characterizations of 7A7 binding to EGFR in human and murine cell lines

**Figure 2:**
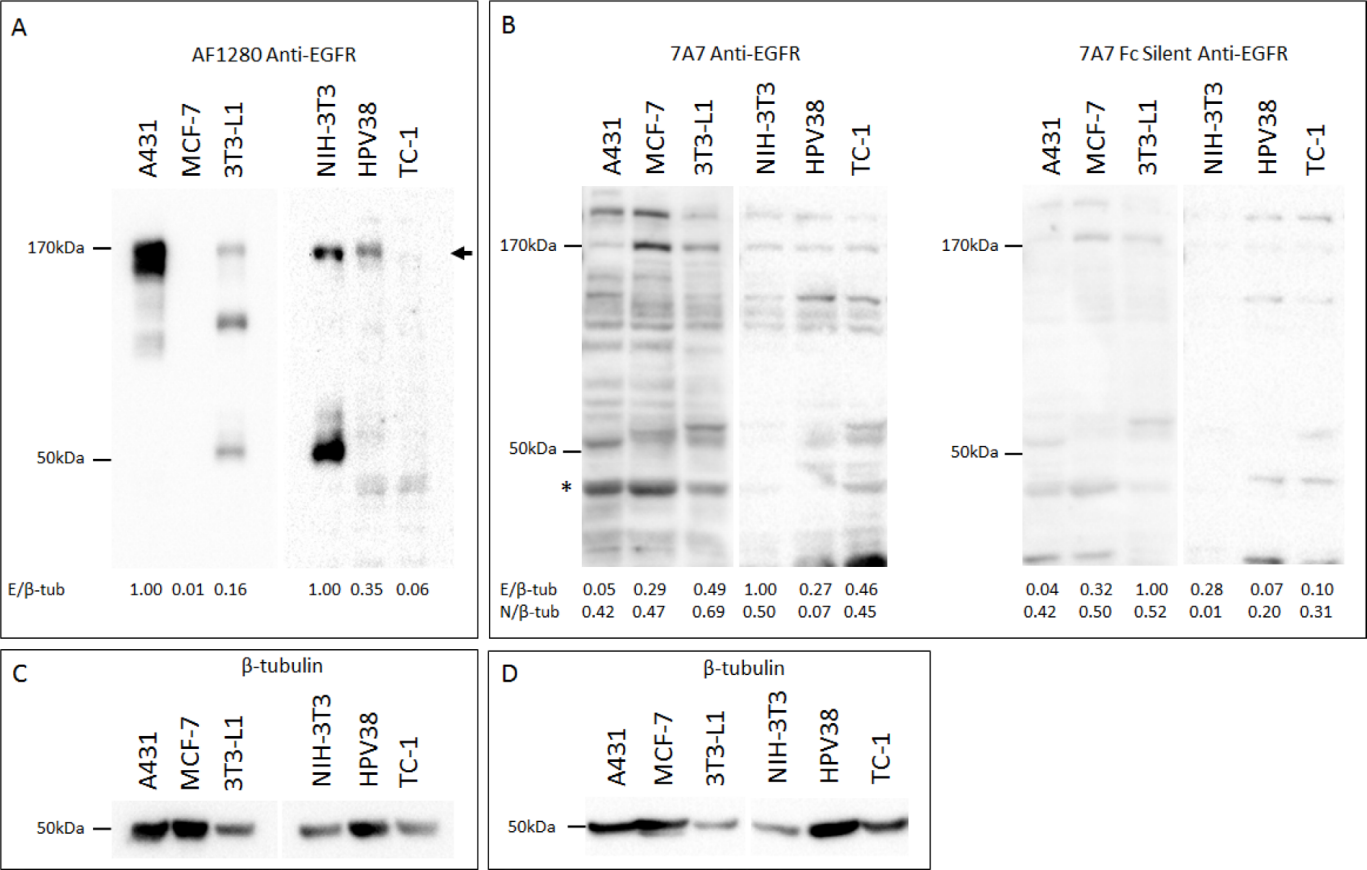
7A7 and 7A7 Fc Silent do not detect EGFR expression by Western blot Protein was extracted from A431, MCF-7, 3T3-L1, NIH-3T3, HPV38, and TC-1 cells, and immunoblotted for EGFR (**A**–**B**) and β-tubulin (**C**–**D**). (A) AF1280; (B) 7A7 and 7A7 Fc Silent; (C) and (D) β-tubulin internal loading controls for (A) and (B) respectively. EGFR was detected as a ∼ 170kDa band by AF1280 (as shown by arrow). Numbers at the bottom of each lane in (A) and (B) represent quantification of each lane normalised to corresponding β-tubulin measurements and relative to the highest resulting ratio on the respective blots. ^*^ = position of non-specific band used as a comparator for EGFR-specificity. E/β-tub; EGFR/β-tubulin, N/β-tub; non-specific band/β-tubulin.

Having validated and identified EGFR positive and negative human and murine cell lines at an RNA level, we evaluated the capacity of 7A7 mAb to detect EGFR protein by SDS-PAGE Western blot (SDS WB). Immunoblotting of human A431 and murine 3T3-L1 and HPV38 protein extracts with the polyclonal goat anti-mouse antibody “AF1280” showed an ∼170 KDa band which corresponded with the predicted size of EGFR (Figure 2A) [17]. A similar band was not detected for TC-1 cells, as expected. However, in our study, we did not observe specific binding of 7A7 or 7A7 Fc Silent mAbs to EGFR by Western blot (Figure 2B; β-tubulin loading controls shown in Figures 2C and 2D). Both 7A7 and 7A7 Fc Silent antibodies bound numerous bands which did not consistently correspond to the molecular weight of EGFR or to the bands seen for AF1280 antibody (see EGFR quantifications in Figures 2A and 2B, and the comparative values following quantification of a non-specific band in Figure 2B). These results indicate that 7A7 and 7A7 Fc Silent mAbs fail to selectively detect both human and mouse EGFR proteins.

**Figure 3:**
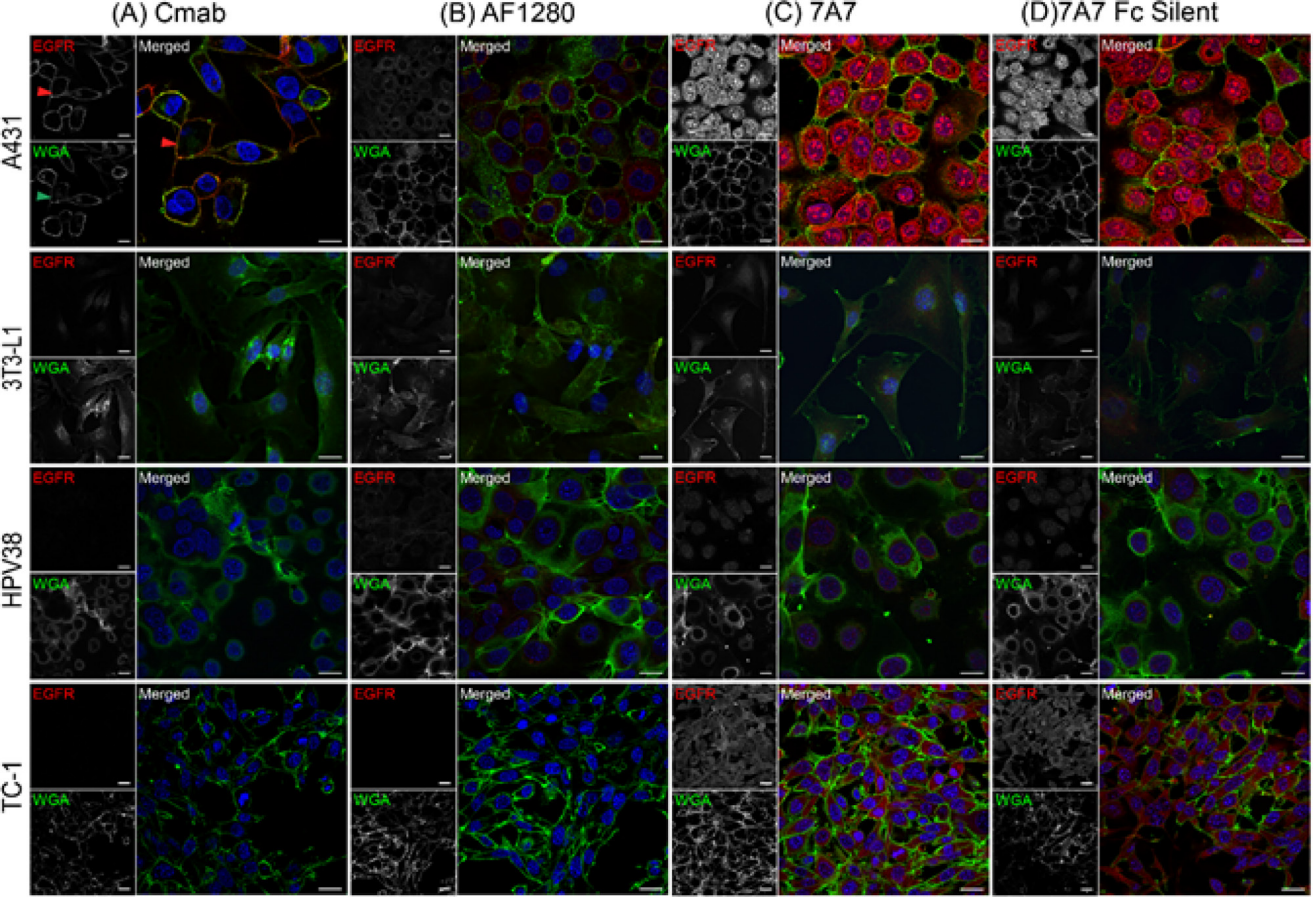
7A7 and 7A7 Fc Silent do not detect EGFR expression by immunofluorescence Four cell lines, A431, 3T3-L1, HPV38 and TC-1 were used to assess the binding of cetuximab (**A**), AF1280 (**B**), 7A7 (**C**) and 7A7 Fc Silent (**D**) antibodies as described in *Materials and Methods*. Red staining: Alexa Fluor 594-conjugated secondary antibodies, Blue staining: DAPI (nucleus), Green staining: plasma membrane as indicated by wheat germ agglutinin (WGA-FITC). Cmab; Cetuximab. Scale bar is 20 µm.

To determine whether 7A7 could still bind EGFR in its native conformation, EGFR localisation was analysed by immunofluorescence and flow cytometry using the human cell line A431 and three murine cell lines 3T3-L1, HPV38, and TC-1. Cetuximab, a human EGFR-specific antibody, immuno-stained EGFR on the plasma membrane in A431 cells (red arrow), as confirmed by its co-localisation with the membrane marker wheat germ agglutinin (WGA, green) (Figure 3A, green arrow) [18]. The cell lines 3T3-L1 and HPV38 express EGFR. Murine EGFR was not detected by the human-specific cetuximab, as expected. The AF1280 antibody detected low levels of EGFR on the plasma membrane of both 3T3-L1 and HPV38 cells (Figure 3B), and high levels of EGFR on the plasma membrane of A431 cells, consistent with levels of mRNA observed in Figure 1. Also consistent with Figure 1, TC-1 cells showed no staining with AF1280 antibody (Figure 3B). Staining of 7A7 and 7A7 Fc Silent were similar between cell lines. Unexpectedly, both 7A7 and 7A7 Fc Silent showed cytoplasmic staining in A431 cells, 3T3-L1 cells, and HPV38 cells, and both plasma membrane and cytoplasmic staining in TC-1 cells, which do not express EGFR (Figure 3C, 3D). The presence of 7A7 and 7A7 Fc Silent immunostaining in the cytoplasm of both EGFR positive and negative cell lines again suggests non-specific binding by 7A7.

**Figure 4:**
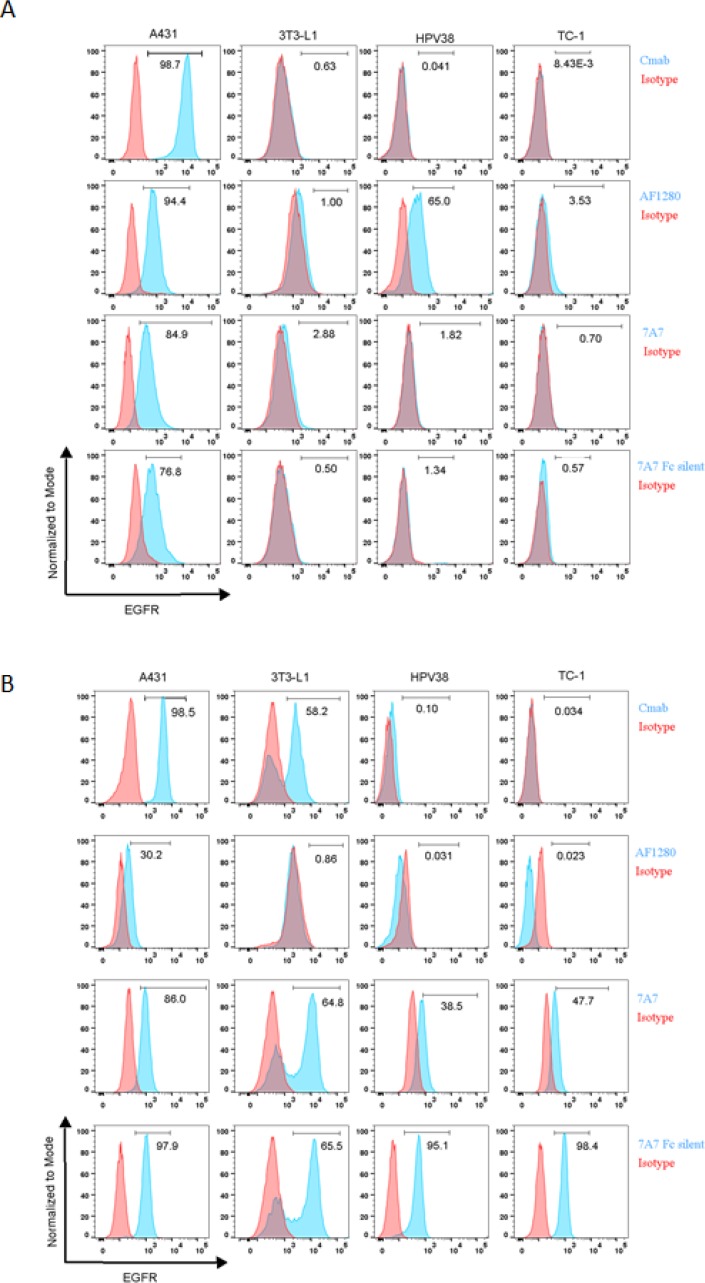
7A7 and 7A7 Fc Silent do not detect EGFR expression by flow cytometry Human (A431) and mouse (3T3-L1, HPV38 and TC-1) cell lines were stained for cell surface EGFR (**A**) or intracellular EGFR (**B**) with cetuximab, AF1280, 7A7 and 7A7 Fc Silent antibodies (blue peaks) or isotype control antibodies (red peaks) as described in *Materials and Methods*.

We also used flow cytometry to examine the capacity of 7A7 to bind to the EGFR. The surface expression of EGFR in A431 cells was successfully detected by both cetuximab and AF1280 antibody (Figure 4A). Interestingly, staining was also detected on the surface of A431 cells by 7A7 and 7A7 Fc Silent (Figure 4A). The AF1280 antibody detected EGFR on the surface of 3T3-L1 and HPV38, however, while 7A7 weakly stained 3T3-L1 cells, 7A7 Fc Silent did not, and neither antibody stained the surface of HPV38 cells (Figure 4A). Similar to our observations by immunofluorescence, only when the three murine cell lines were permeabilised to investigate intracellular protein production did 7A7 and 7A7 Fc Silent antibodies produce consistent staining (Figure 4B). Taken together, these data suggest that 7A7 and 7A7 Fc Silent bind to an unknown intracellular protein(s) which do not correspond to human nor murine EGFR.

### EGFR immunostaining in tumour samples

**Figure 5:**
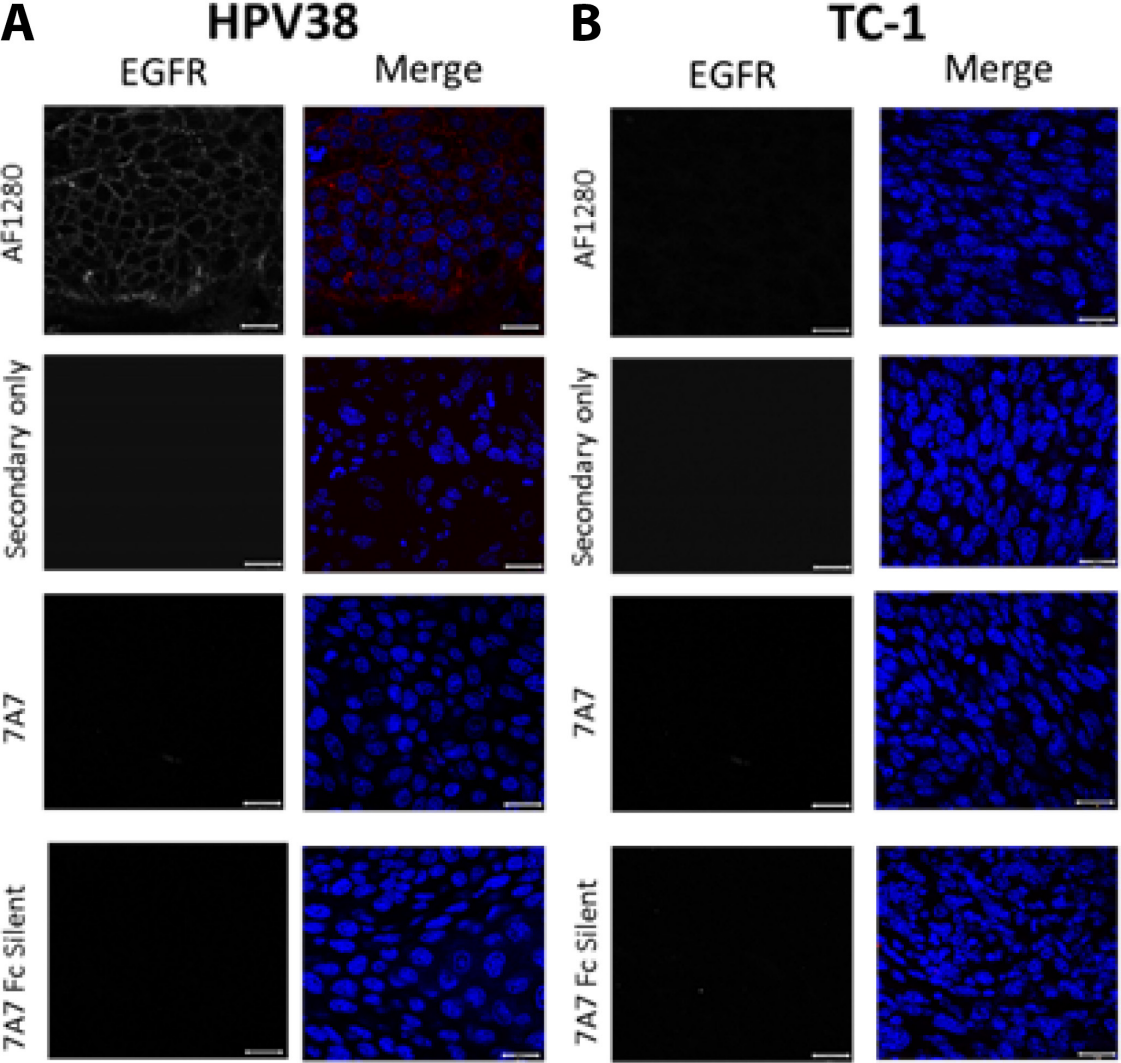
7A7 and 7A7 Fc Silent do not detect EGFR expression in HPV38 tumour tissue by immunofluorescence EGFR expression in formalin fixed paraffin-embedded samples from HPV38 (**A**) and TC-1 (control; **B**) tumour tissues. Sections were immunostained with 3 primary antibodies (7A7, 7A7 Fc Silent and AF1280) and Alexa Fluor 594-conjugated anti-mouse or anti-goat secondary antibodies (red). Secondary only (Alexa^594^-conjugated goat anti-mouse IgG1 or Alexa^594^-conjugated donkey anti-goat IgG1) served as negative controls. Nuclei were stained using DAPI (blue). Scale bar is 20 µm.

Next, we wanted to assess EGFR expression and localisation in our preclinical mouse models. We harvested HPV38 and TC-1 tumours growing in mice, and examined the expression of EGFR in paraffin-embedded sections by immunofluorescence (Figure 5). We demonstrated that the AF1280 antibody could successfully detect EGFR on the plasma membrane of HPV38 tumour sections but not in TC-1 derived tumours (Figure 5A–5B). In contrast, 7A7 and 7A7 Fc Silent antibodies failed to detect EGFR protein expression on the same tumour samples.

### *In vivo* administration of 7A7 mAb did not affect tumour growth in subcutaneous TC-1 and HPV38 tumours

**Figure 6:**
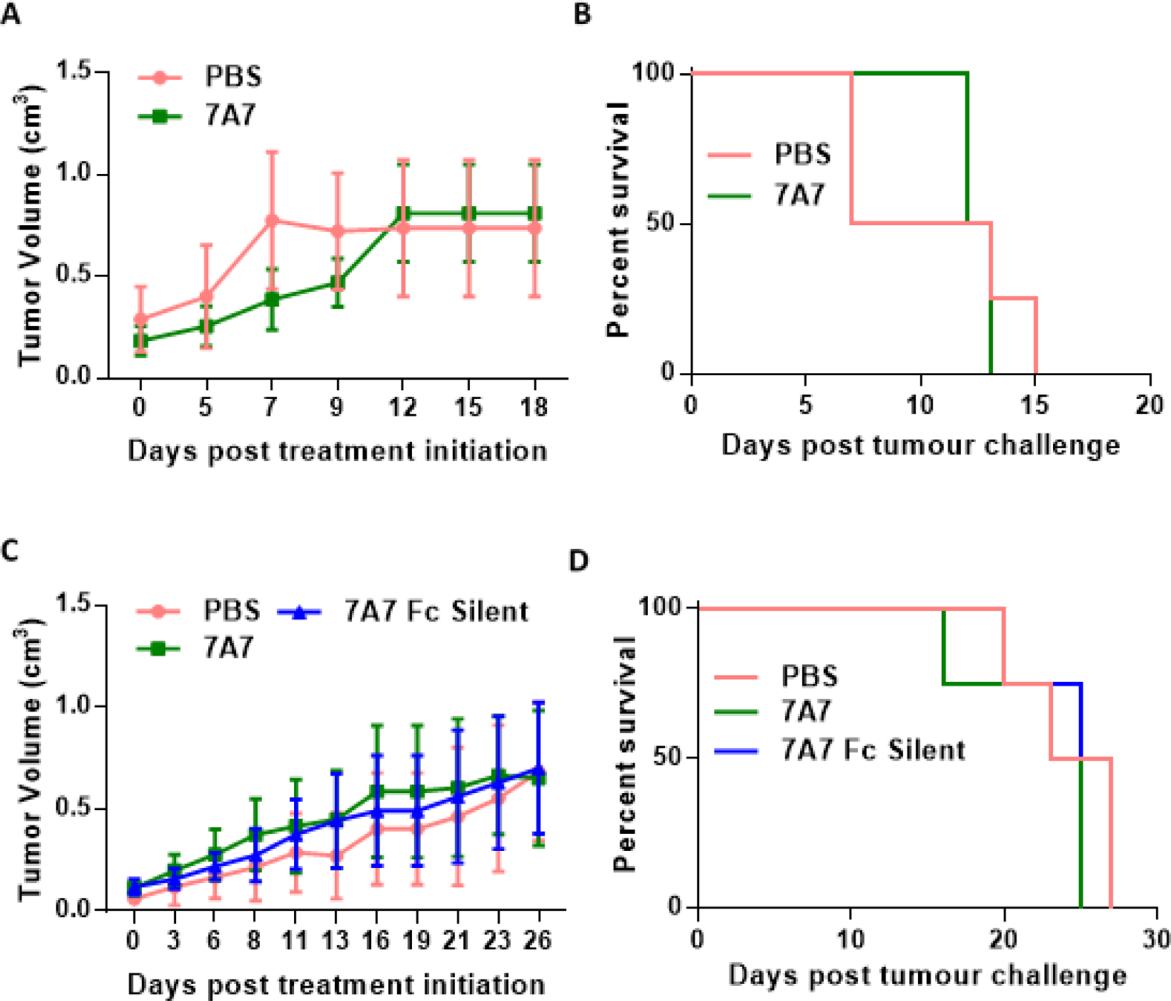
7A7 does not affect tumour growth *in vivo* Established TC-1 (**A**–**B**) or HPV38 (**C**–**D**) tumours were treated with PBS (i.v.) or 56ug of 7A7 (i.v.) or 7A7 Fc Silent (i.v.) every three days. (A, C) Tumour growth curves, and (B, D) survival curves. (A, B) *n* = 4-6 mice/group; (C–D) *n* = 4 mice/group. Data shown represent two independent experiments. (A-D) No statistical differences between groups.

Our experiments indicate that 7A7 does not detect mouse EGFR *in vitro*. To investigate the ability of 7A7 to target EGFR and modulate tumour growth *in vivo*, we used 7A7 to treat mice with established HPV38 or TC-1 tumours. Mice were challenged with viable HPV38 or TC-1 cells, and after tumours reached 0.2-0.3 cm^3^ in size, the mice were treated every three days with either 7A7 or PBS (or 7A7 Fc Silent – HPV38 tumours only). No significant differences in TC-1 growth (Figure 6A) nor survival (Figure 6B) were observed following treatment, as predicted. Similarly, in the HPV38 tumour model, no significant differences in tumour growth (Figure 6C) nor survival (Figure 6D) were observed between treatments. Cumulatively, these results required us to reconsider 7A7 as a model for mAb therapy targeted against EGFR in mice.

## DISCUSSION

EGFR is considered to be an important target for multiple cancer therapies [19, 20]. Currently, two different types of EGFR targeted drugs are used in the clinic, therapeutic mAbs which can block the extracellular ligand-binding domain and induce ADCC, and small molecule inhibitors targeting the EGFR signaling cascade [21]. The vast majority of commercially-available EGFR-targeted antibodies are specific for human EGFR. Thus, preclinical data is normally derived from human tumour xenograft models in immune-suppressed mice [22]. However, to fully understand the characteristics of these EGFR targeted drugs the role of the immune system needs to be considered. This could be assessed in preclinical mouse tumour models in the context of an active immune system. Therefore, there is a necessity for proper anti-mouse EGFR antibody models to be developed in mice to enable these preclinical studies.

7A7, an anti-mouse EGFR IgG1 mAb, was first published by Garrido *et al.* in 2004 [13]. The authors demonstrated 7A7 could recognise mouse EGFR and was cross-reactive with the human EGFR. Additionally, they found that 7A7 could inhibit EGF-induced EGFR signaling in D122 tumour cells, triggering tumour regression in a T-cell dependent manner [14, 23]. Thus, it was suggested that 7A7 was similar to cetuximab and an ideal anti-mouse EGFR mAb candidate for preclinical studies. We therefore chose to pursue this antibody as a model to establish pre-clinical studies.

This is the first unbiased study of 7A7 and 7A7 Fc Silent mAbs and their potential anti-tumour capacity. The *in vitro* specificity of 7A7 mAb and 7A7 Fc Silent, which are produced by different hybridomas, was assessed by WB, IF and FACS in different human and mouse cell lines. We found that neither 7A7 nor 7A7 Fc Silent could detect human nor murine EGFR by SDS-WB, however both antibodies were able to bind to some unknown proteins inside the cell as determined by SDS-WB, IF and FACS techniques. The specificity of the 7A7 mAb to bind EGFR expressed by *in vivo* tumours was studied in HPV38 and TC-1 (control) tumour samples. However, in contrast to staining with our positive control AF1280 antibody, 7A7 failed to detect EGFR expression on EGFR^+^ HPV38 tumour sections by IF. This is unlikely to be due to batch issues as 7A7 mAb and 7A7 Fc Silent are produced by different hybridomas and colleagues at other institutes discussed similar issues with 7A7 (personal communications).

Lastly, we characterized the anti-tumour capacity of 7A7 in *in vivo* TC-1 and HPV38 tumour models. We followed the dose and dosing schedule outlined in the original papers describing the *in vivo* use of 7A7 [14, 23]. In contrast to our data, Garrido et al. showed that 7A7 could detect mouse EGFR successfully on the TC-1 cell line *in vitro*. Interestingly however, the TC-1 tumour was reported to be insensitive to 7A7 treatment, with the escape mechanisms not identified [24]. A second cell line, 3LL-D122, was also reported to express EGFR, but was responsive to 7A7 treatment [24], suggesting that EGFR expression levels in these two murine cell lines were not directly indicative of treatment response to 7A7. In our hands, we could not demonstrate that TC-1 tumour cells express EGFR, but we also found that 7A7-treated mice bearing TC-1 tumours failed to show tumour regression or a halt in growth when compared to PBS-injected mice. However, the same was true for HPV38 tumours, which we were able to confirm express EGFR by multiple methods. In conclusion, contrary to the findings of previously published studies [13, 14, 22, 25–27], our results did not find any evidence to support that 7A7 is able to recognise mouse or human EGFR on either cell lines or tumour tissue.

The reason of this discrepancy is unclear. However, aside from the laboratory that first reported 7A7, we note that there are no other publications in the literature that have used this mAb. Ideally, a direct comparison should be made using the 3LL-D122 cell line, which is a highly metastatic clone of 3LL, however this cell line is not widely available and we have been unable to obtain it. Upon receipt of 7A7 and 7A7 Fc Silent we ascertained that there was antibody in the tubes by measuring the protein concentration and running a sample of the antibody on an SDS-PAGE gel and subsequently performing Coomassie staining; both the heavy and light chain of the antibodies appeared intact and undegraded. We can’t know whether the batch of 7A7 used in the study was faulty/damaged, however we have compared 7A7 with 7A7 Fc-silent, which is produced by a different hybridoma and is therefore a “different batch”. 7A7 Fc-silent has been modified at the Fc antibody region so that it will not bind to Fc receptors and induce antibody-dependent cellular cytotoxicity, however it should still bind to the EGFR by its antigen-binding domains and this should be detectable. However, we could not demonstrate EGFR binding by 7A7 OR 7A7 Fc silent. Unfortunately, there is not an alternative supplier of 7A7 and the original authors have yet to respond to our requests for a sample of 7A7. A further experiment that could be performed to assess the specificity of 7A7 is to transfect murine Egfr into a cell line as a positive control for Egfr expression. However, since our study shows that 7A7 recognised many bands in both Egfr positive and Egfr negative cell lines and was mislocalised by immunofluorescence, it seems likely that non-specific binding is occurring. Based on our data we conclude that 7A7 does not detect mouse EGFR and it is not an ideal mouse version of cetuximab for preclinical studies.

## MATERIALS AND METHODS

### Mice

C57BL/6 mice were purchased from the Animal Resources Centre (Perth, Australia). HPV38E6E7 FVB x Rag1 KO mice were bred and maintained at the Translational Research Institute Biological Research Facility (Brisbane, Australia). All mice were 6–12 week females housed under specific pathogen-free conditions. Animal procedures were approved by the University of Queensland Animal Ethics Committee.

### Cell lines

The HPV38 SCC cell line was established in our laboratory following the UV-induction of an SCC tumour in an HPV38E6E7-FVB transgenic mouse [28]. HPV38 cells were cultured in modified Ham’s F12 media (Thermo Fisher Scientific, Waltham, MA, USA). Modified Ham’s F12 media: 25% Dulbecco’s Modified Eagle’s Medium (DMEM)/high glucose (Thermo Fisher Scientific), 5% fetal bovine serum (FBS, Thermo Fisher Scientific), 5µg/mL insulin (Sigma-Aldrich, Castle Hill, NSW, Australia), 0.4 µg/mL hydrocortisone (Sigma-Aldrich), 10 ng/mL human recombinant epidermal growth factor (Invitrogen, Carlsbad, CA), 8.4 ng/mL Cholera toxin from *Vibrio cholera* (Sigma-Aldrich), 24 µg/mL adenine (Sigma-Aldrich) and 1X penicillin/streptomycin/glutamine (Life Technologies, Carlsbad, CA). TC-1 cells were maintained in RPMI1640 (Life Technologies), containing 20% FBS. A431, MCF-7, NIH-3T3, and 3T3-L1 cell lines were maintained in DMEM containing 10% FBS. All cells were grown at 37°C in 5% CO_2_.

### Quantitative real time PCR (q-PCR)

Total RNA isolated from cells was subjected to cDNA synthesis and q-PCR using iQ SYBR-Green Supermix as per manufacturer’s instructions (Invitrogen). cDNA reverse reactions were incubated under the following conditions: 65°C 5 mins, 4°C 1 min, 25°C 5mins, 50°C 60 mins, 70°C 15 mins, 4°C∞.The mRNA levels were normalized to that of the housekeeping gene, hypoxanthine guanine phosphoribosyltransferase (HPRT) based on the threshold cycle (Ct) of each sample in RT q-PCR. Relative levels of mRNA expression were calculated from ΔCt where ΔCt = (test mRNA Ct- HPRT Ct). The forward and reverse primers (5**′**–3**′**) used were as follows: mEgfr forward: TCTTCAAGGATGTGAAGTGTG; mEgfr reverse: TGTACGCTTTCGAACAATGT; hEGFR forward: GCCAAGGCACGAGTAACAAGC; hEGFR reverse: AGGGCAATGAGGACATAACC.

### Western blot analysis

Cell lysates were prepared in RIPA buffer (1% Nonidet P-40, 0.5% sodium deoxycholate, 0.1% SDS in 10mM Tris-HCl pH7.4) with protease and phosphatase inhibitor set (CatLog# 539134, Calbiochem, Merck KGaA, Darmstadt, Germany). Protein concentrations were determined using the Pierce™ BCA protein assay kit (Thermo Fisher Scientific) according to the manufacturer’s instructions. Cell extracts were applied to 8% SDS-PAGE gels and transferred to polyvinylidinedifluoride membranes (PVDF; Sigma-Aldrich). PVDF membranes were blocked with 2% BSA in PBS and incubated with 7A7 (mouse, Absolute Antibody, Oxford, UK), 7A7 Fc Silent (mouse, Absolute Antibody), anti-mouse EGFR “AF1280” (polyclonal goat antibody, R&D Systems, New South Wales, Australia) or β-tubulin (clone 2-28-33, Invitrogen) primary antibodies and anti-mouse or anti-goat HRP secondary antibodies (Invitrogen). Membranes were incubated with Chemiluminescent Substrate (Bio-rad, Gladesville, Australia) and imaged using the ChemiDoc XRS+ System with Image Lab™ software (Bio-rad).

### Immunofluorescence

Cells were seeded onto coverslips in 6-well plates and grown in culture medium for 24h. Cells were incubated with wheat germ agglutinin (WGA-FITC) (Life Technologies), then fixed with 4% paraformaldehyde for 30mins, washed with PBS and permeabilised with 0.1% Triton-X100 (Sigma-Aldrich) for 10 mins. Coverslips were blocked with 2% BSA/PBS and then incubated with 7A7, 7A7 Fc Silent, cetuximab (Merck) or AF1280 primary anti-EGFR antibodies diluted in 2% BSA/PBS for 60 mins at room temperature. After washing with PBS, cells were incubated with Alexa Fluor 594-conjugated anti-mouse, anti-human or anti-goat IgG antibodies (Life Technologies), respectively. Samples were imaged using the Zeiss 510 meta confocal microscope (Zeiss, Lonsdale, Australia) and images were processed using Image J and Adobe illustrator.

### Immunofluorescence of paraffin-embedded sections

EGFR expression was analysed in formalin-fixed paraffin embedded tumour sections as described below. Sections were deparaffinised in xylene (twice for 5 minutes) and rehydrated with alcohol (twice in 100% alcohol for 5 minutes, and then in 90%, 80%, 70%, 50%, 30% for 1 minute each). Antigen retrieval was performed using 20ul/ml proteinase K solution for 10 min at 37°C. Sections were then washed with PBS and blocked with 10% mouse serum in PBS for 30 minutes. Sections were then incubated with either 7A7, 7A7 Fc Silent, cetuximab or AF1280 antibody overnight at 4°C. After washing with PBS, sections were incubated with Alexa-Fluor-594 conjugated anti-mouse, anti-human or anti-goat secondary antibodies (Life Technologies) respectively. Samples were imaged using the Zeiss 510 meta confocal microscope (Zeiss, Lonsdale, Australia) and images were processed using Image J.

### Flow cytometry

Cells were prepared for flow cytometry by washing with PBS and then FACs buffer (2% FBS in PBS). Cell suspensions (1x10^6^cells) were stained with 7A7, 7A7 Fc Silent, cetuximab or AF1280 antibodies, and incubated with Alexa 488-conjugated-anti-mouse, anti-human or anti-goat secondary antibodies (Life Technologies) respectively. For intracellular staining, fixation and permeabilisation of cells was performed using a Fix & Perm cell permeabilisation kit (eBioscience, Thermo Fisher Scientific) according to the manufacturer’s instructions. Samples were acquired on a Fortessa X20 flow cytometer (BD Bioscience, New Jersey, USA) and data analyzed using Flow Jo 10.0 software (Treestar, San Carios, CA, USA).

### Tumour transplantation and antibody treatment

1x10^6^ TC-1 cells or 1x10^6^ HPV38 cells were injected subcutaneously into C57BL/6 mice or HPV38E6E7 x Rag1 KO mice respectively, in 100ul of PBS solution. Once tumours had grown to 0.2-0.3cm^3^ the animals received PBS (i.v.), 7A7 (56 µg, i.v.), or 7A7 Fc Silent (56 µg, i.v.) every three days to experimental endpoint as per respective groups. Tumour growth was recorded three times per week.

### Statistical analysis

All statistical analysis was carried out using GraphPad Prism version 6.02 or 7.03 (GraphPad Software, San Diego, CA, USA). Statistical analysis of RT-PCR data was performed using an unpaired student’s *t*-test or a one-way ANOVA with post-hoc Tukey’s multiple comparisons test, and statistical analysis of mouse survival data was performed using a Log-rank (Mantel-Cox) test. A *p* value of *p* < 0.05 (^*^) was considered significant. *p* < 0.0001 (^****^) is also indicated.
